# N-WASP Attenuates Cell Proliferation and Migration through ERK2-Dependent Enhanced Expression of TXNIP

**DOI:** 10.3390/biology11040582

**Published:** 2022-04-11

**Authors:** Yat Joong Chung, Amrita Salvi, Pazhanichamy Kalailingam, Myra Alnawaz, Suat Hoon Tan, Jiun Yit Pan, Nguan Soon Tan, Thirumaran Thanabalu

**Affiliations:** 1 School of Biological Sciences, Nanyang Technological University, 60 Nanyang Drive, Singapore 637551, Singapore; chun0059@e.ntu.edu.sg (Y.J.C.); amritas@uic.edu (A.S.); pazhanichamy.k@ntu.edu.sg (P.K.); myra.alnawaz@ntu.edu.sg (M.A.); nstan@ntu.edu.sg (N.S.T.); 2 National Skin Centre, 1 Mandalay Road, Singapore 308205, Singapore; shtan@nsc.com.sg (S.H.T.); jypan@nsc.com.sg (J.Y.P.); 3 Lee Kong Chian School of Medicine, Nanyang Technological University, 11 Mandalay Road, Singapore 308232, Singapore

**Keywords:** cell adhesion, actin cytoskeleton, skin cancer, proteasome, protein microarray

## Abstract

**Simple Summary:**

Neural Wiskott–Aldrich Syndrome Protein (N-WASP) regulates actin cytoskeleton remodeling and can, it has been suggested, suppress several cancers. In this study, HSC-5 cells, a mammalian cell line with reduced N-WASP expression, were used to generate control cells and HSC-5 cells with increased N-WASP expression that is comparable to that of normal keratinocytes. The two cell lines were used to elucidate the regulation of cell proliferation and migration by N-WASP. Our findings suggest that N-WASP increases ERK2-dependent phosphorylation of FOXO1 and increases TXNIP expression, which reduces cell proliferation and migration. This study is the first to propose an antiproliferative role of N-WASP, which is mediated via ERK2, and it suggests new avenues for cancer therapeutic research and treatment.

**Abstract:**

Neural Wiskott–Aldrich Syndrome Protein (N-WASP) regulates actin cytoskeleton remodeling. It has been known that reduced N-WASP expression in breast and colorectal cancers is associated with poor prognosis. Here, we found reduced N-WASP expression in squamous cell carcinoma (SCC) patient samples. The SCC cell line HSC-5 with reduced N-WASP expression was used to generate HSC-5^CN^ (control) and HSC-5^NW^ (N-WASP overexpression) cells. HSC-5^NW^ cells had reduced cell proliferation and migration compared to HSC-5^CN^ cells. HSC-5^NW^ cells had increased phospho-ERK2 (extracellular signal-regulated kinase 2), phosphorylated Forkhead box protein class O1 (FOXO1) and reduced nuclear FOXO1 staining compared to HSC-5^CN^ cells. Proteasome inhibition stabilized total FOXO1, however, not nuclear staining, suggesting that FOXO1 could be degraded in the cytoplasm. Inhibition of ERK2 enhanced nuclear FOXO1 levels and restored cell proliferation and migration of HSC-5^NW^ to those of HSC-5^CN^ cells, suggesting that ERK2 regulates FOXO1 activity. The expression of thioredoxin-interacting protein (TXNIP), a FOXO1 target that inhibits thioredoxin and glucose uptake, was higher in HSC-5^NW^ cells than in HSC-5^CN^ cells. Knockdown of TXNIP in HSC-5^NW^ cells restored cell proliferation and migration to those of HSC-5^CN^ cells. Thus, we propose that N-WASP regulates cell proliferation and migration via an N-WASP-ERK2-FOXO1-TXNIP pathway.

## 1. Introduction

The skin consists of the epidermis and the dermis, which are separated by a basement membrane. It is the largest organ of the human body and protects all constituent organs from foreign pathogens [1]. Skin cancer is the most common human cancer and is divided into melanoma and non-melanoma skin cancer (NMSC). NMSC is further divided into basal cell carcinoma (BCC) and squamous cell carcinoma (SCC) [2]. Mutations caused by ultraviolet (UV) radiation are the leading cause of BCC and SCC [2]. SCCs are generally more fatal than BCCs as they develop from multiple tumor foci with high metastatic and proliferative activities [3]. Mutations in the tumor protein 53 (TP53) and phosphoinositide 3-kinase/protein kinase B (PI3K/AKT) pathways and upregulations of α5β1 and αvβ6 integrins, cellular homolog of retroviral v-Myc oncogene (c-Myc, Src-family tyrosine kinases and epidermal growth factor receptor (EGFR)-mediated signaling have all been implicated in SCC development [4,5].

The actin cytoskeleton, an intracellular protein network, is critical for maintaining cell shape and supporting cellular processes such as cell division and migration [6]. Regulation of actin recycling and polymerization of globular G-actin into filamentous F-actin is essential for actin cytoskeleton remodeling and thus critical for cellular processes that rely on the actin cytoskeleton [7]. Actin cytoskeleton remodeling is regulated by actin nucleation factors, such as the actin-related protein 2/3 (Arp2/3) complex. The Arp2/3 complex is in turn controlled by nucleation-promoting factors, such as members of the Wiskott–Aldrich syndrome protein (WASP) family, including WASP, WASP-family verprolin-homologous protein 1 (WAVE) and neural WASP (N-WASP) [8,9,10,11]. The expression of N-WASP is reduced in breast and colorectal cancers and correlates with poor prognosis for patients with these cancers [12,13]. N-WASP has also been implicated in lung cancer metastasis [14]. These observations suggest that N-WASP may play critical roles in multiple cancers. However, the role of N-WASP in skin cancer has not been reported. We recently showed that conditional knockout of N-WASP in mouse keratinocytes caused epidermal hyperplasia due to the increased proliferation in keratinocytes [15], suggesting that N-WASP might play a negative role in keratinocyte proliferation and skin carcinogenesis.

Transcription factors (TFs) regulate gene expression [16,17], and mutations in TFs may cause aberrant expression of genes, which can lead to abnormal growth and cancer development [18]. Forkhead box class O protein 1 (FOXO1) is a member of the FOX family of TFs that regulates cell metabolism, growth, and proliferation [19]. FOXO1 contains a forkhead box DNA-binding domain, a nuclear localization sequence, a nuclear export sequence, and a transactivation domain [20]. It is regulated via phosphorylation by kinases such as AKT, serum and glucocorticoid-regulated kinase (SGK1), and c-Jun N-terminal kinase (JNK), resulting in the export of FOXO1 from the nucleus to the cytoplasm and subsequent proteasomal degradation [20,21]. Extracellular signal-regulated kinase 2 (ERK2) has been reported to phosphorylate FOXO1 at many residues in human cancer cells [22]. FOXO1 has been shown to play a role in skin epidermal morphogenesis and repair and regulates basal keratinocyte cell proliferation [23,24].

Oxygen is vital for aerobic cellular metabolism. However, reactive oxygen species (ROS), formed from metabolic processes and stimuli, such as UV radiation [25], cause oxidative stress that is detrimental to cells [26]. The highly conserved thioredoxin system scavenges excess intracellular ROS by reducing them to H_2_O [25]. Thioredoxin-interacting protein (TXNIP), previously called thioredoxin-binding protein 2 (TBP-2) and vitamin D3-upregulated protein 1 (VDUP1) [27,28], is a ubiquitously expressed protein that negatively regulates thioredoxin-mediated scavenging of ROS and causes ROS build-up [29]. TXNIP, an α-arrestin family protein, has been shown to suppress glucose uptake by binding to the receptor glucose transporter 1 (GLUT1) and inducing GLUT1 internalization [30]. The expression of TXNIP is negatively regulated by FOXO1 [31], and TXNIP has been implicated in the inhibition of cell proliferation and promotion of apoptosis by activating apoptosis signal-regulating kinase 1 (ASK1)-dependent signaling [32]. The overexpression of TXNIP in NIH3T3 cells reduced thioredoxin levels and increased intracellular ROS [33]. The treatment of metastatic neuroblastomas with fenofibrate, an antihyperlipidemic drug, increased TXNIP levels and repressed cell proliferation and migration [34]. In contrast, TXNIP knockdown or loss-of-function mutation coincided with cancerous development [35].

In this study, we found that SCC samples had reduced N-WASP expression compared to the matched perilesional samples. HSC-5 cells, a skin SCC cell line, have reduced N-WASP levels. Ectopic overexpression of N-WASP in HSC-5 cells reduced cell proliferation and migration compared to those of HSC-5^CN^ cells. HSC-5^NW^ cells have more junctional E-cadherin than HSC-5^CN^ cells, suggesting that the reduced cell migration of HSC-5^NW^ cells is probably due to increased cell-cell and cell-ECM adhesion. HSC-5^NW^ cells have altered phospho-FOXO1 levels, reduced nuclear FOXO1, and increased the expression in TXNIP. The inhibition of ERK2 increased nuclear FOXO1 localization and restored the proliferation and migration of HSC-5^NW^ cells to those of HSC-5^CN^ cells. Similarly, knocking down TXNIP in HSC-5^NW^ cells restored cell proliferation and migration to those of HSC-5^CN^ cells. Thus, our results suggest that increased N-WASP levels enhanced ERK2-dependent FOXO1 phosphorylation, the proteasomal degradation of FOXO1, and the expression of TXNIP, leading to the attenuation of cell proliferation and migration in HSC-5 cells.

## 2. Materials and Methods

### 2.1. Cell Culture and Generation of Stable Sublines by Lentiviral Transduction

Cell lines (HSC-5, HaCaT, A549, A431 and HEK293T) were cultured in complete Dulbecco’s modified Eagle’s medium (DMEM with 10% fetal bovine serum (FBS), 1% penicillin and 1% streptomycin) at 37 °C in a humidified incubator with 5% carbon dioxide (CO_2_). TXNIP short hairpin ribonucleic acid (shRNA) (5′-GAA TAT TCA ACT CGA AGG ATG) was cloned into the lentiviral plasmid pLJM1 (a gift from David Sabatini) (Addgene plasmid #19319) [36] under a U6 promoter and used for knocking down TXNIP. Human N-WASP was cloned into the lentiviral plasmid pLJM1 and used for overexpression of N-WASP. Infectious third generation lentiviruses were generated by transfecting HEK293T cells with the target vector together with packaging plasmids (VSVG, REV and pDNL) and harvesting the culture supernatant with virus particles. HSC-5^CN^ and HSC-5^NW^ stable sublines were generated by infecting HSC-5 cells with lentivirus generated with an empty target vector (pLJM1) and a target vector expressing N-WASP, respectively, and a subsequent selection with 2 µg/mL puromycin (Sigma, St. Louis, MO, USA,); this was similarly performed on A431 and A549 cells. HSC-5^NW^ cells were additionally subjected to limited dilutions to isolate and select a clone that expressed N-WASP protein levels similar to those of HaCaT cells. Similarly, TXNIP knockdown cells were generated by infecting cells with lentivirus generated with a target vector expressing TXNIP shRNA and selected with 500 µg/mL neomycin (G-418, Santa Cruz Biotech, Santa Cruz, CA, USA).

### 2.2. Cell Proliferation Assay

Cells were first trypsinized. Next, 7.5 × 10^3^ viable cells, quantified with the Trypan Blue exclusion method, were seeded in 24-well culture plates and incubated for 5 days. After that, they were trypsinized and resuspended in 1 mL of DMEM, and the number of viable cells was counted using a hemocytometer (GMBH, Miami, FL, USA).

### 2.3. Wound Healing Assay

Cells (1 × 10^6^ viable cells/well) were seeded in 6-well culture plates and grown to 100% confluency, and a scratch was made with a sterile 10 µL micropipette tip. The wells were washed once with 1× phosphate-buffered saline (PBS) solution and incubated in complete DMEM. Images were acquired at 0 and 12 or 24 h post-scratch with an Olympus IX51 microscope (Olympus, Breinigsville, PA, USA) fitted with the Cool SNAP^HQ^ camera (Photometrics, Tucson, AZ, USA) and MetaVue program (Molecular Devices, San Jose, CA, USA). The wound area was quantified with ImageJ software (version 1.48) [37].

### 2.4. Western Blot Analysis

Cells were resuspended in radioimmunoprecipitation assay (RIPA) lysis buffer (50 mM Tris-hydrochloric acid (Tris-HCl), 200 mM sodium chloride (NaCl), 1% Triton X-100, 0.1% sodium dodecyl sulfate (SDS), 0.5% sodium deoxycholate, 10% glycerol, 1 mM ethylenediaminetetraacetic acid (EDTA) and 1 mM phenylmethylsulfonyl fluoride (PMSF)) supplemented with 1 mM phosphatase inhibitor sodium orthovanadate (Na_3_VO_4_) (Sigma, St. Louis, MO, USA). The protein concentrations of all cell lysates were determined using a Bradford protein assay kit (BioRad, Hercules, CA, USA), and equal amounts of protein were resolved using 10% SDS–PAGE and transferred onto a 0.45 µm nitrocellulose membrane (BioRad). The membranes were probed with antibodies against N-WASP (laboratory-generated), E-cadherin (BD Transduction, NJ, USA), pan-ERK1/2, phospho-ERK1/2, pan-AKT1/2/3, phospho-AKT1/2/3 (Cell Signaling, Danvers, MA, USA), Cyclin D1, FOXO1, and TXNIP (Santa Cruz Biotech, Santa Cruz, CA, USA), with GAPDH (Ambion, Austin, TX, USA) as a loading control. After incubation with appropriate HRP-conjugated secondary antibodies (Sigma), the membranes were developed using the Immobilon Western Chemiluminescent HRP Substrate (Millipore, Burlington, MA, USA), and digital images were acquired using the LAS-4000 apparatus (Fujifilm, Tokyo, Japan). Protein band intensities were then quantified with ImageJ.

### 2.5. Immunofluorescence

Cells seeded in a 6-well culture plate with a coverslip were fixed with 3.7% paraformaldehyde, permeabilized with 0.2% Triton X-100 in 1× PBS, and blocked with 1% BSA in 1× PBS. The cells were probed with the antibodies anti-E-cadherin (BD Transduction) and anti-FOXO1 (Santa Cruz Biotech, Santa Cruz, CA, USA) (1°), followed by incubation with Alexa Fluor 488-conjugated secondary antibody (2°) and Alexa Fluor 568 phalloidin (Molecular Probes, Eugene, OR, USA). Cells were also incubated with DAPI (Molecular Probes) for nuclear staining. Images of stained cells were acquired using an Olympus IX51 microscope fitted with the Cool SNAP^HQ^ camera and MetaVue program. The E-cadherin fluorescence intensity was quantified using a freehand icon in the ImageJ program to draw regions of interest (ROIs) for all cell membranes of a randomly chosen cell. The fluorescence was quantified. The values of the ROI were divided by 2 if the ROI covered cell membranes from 2 cells, and these values were then averaged. The average fluorescence intensity of 20 randomly chosen cells was determined. Similarly, FOXO1 fluorescence intensity was quantified by using a freehand icon in ImageJ to draw an ROI for the nucleus of a randomly chosen cell, the fluorescence was quantified, and the average fluorescence intensity of 20 randomly chosen cells was determined. The quantifications were performed on 3 independent assays.

### 2.6. Kinex^TM^ Antibody Microarray Analysis

HSC-5^CN^ and HSC-5^NW^ cells grown in 10-cm culture dishes were scraped, washed with 1× PBS, lysed, labeled with fluorophores, and incubated at room temperature in the Kinex KAM-880 Antibody Microarray and processed according to the manufacturer’s protocol (Kinexus Bioinformatics, Vancouver, BC, Canada). The microarray was imaged using the Gene Pix Pro 6.0 program and the GenePix 4000B Microarray Scanner (Molecular Devices, San Jose, CA, USA) using the KAM-880 microarray format.

### 2.7. Real-Time PCR

Mammalian cells were lysed, and total ribonucleic acid (RNA) was extracted using TRIzol according to the manufacturer’s protocol (Invitrogen, Waltham, MA, USA). Total RNA from paraffin-embedded in vivo SCC samples and matched perilesional samples was isolated, briefly, by the dissolution of paraffin in xylene, removal of proteins with proteinase K digestion, further incubation of samples at 80 °C for reversal of formalin crosslinking, and digestion of genomic DNA with DNase and then eluted according to the manufacturer’s protocol (Qiagen, MD, USA). Then, 2 µg of RNA was reverse-transcribed to complementary deoxyribonucleic acid (cDNA) and was used as a template in a real-time polymerase chain reaction (PCR) with the SYBR Green Master Mix (Fermentas, Waltham, MA, USA) and Applied Biosystems 7500 Real-Time PCR system (Applied Biosystems, Bedford, MA, USA). The sense and antisense primer sequences were as follows: human N-WASP, 5′-AAG GAT GGG AAA CTA TTG TGG GA and 5′-GAC GGC CCA AAA GGT CTG TAA; human FOXO1, 5′-TTA TGA CCG AAC AGG ATG ATC TTG and 5′-TGT TGG TGA TGA GAG AAG GTT GAG; human TXNIP, 5′-ACT CGT GTC AAA GCC GTT AGG and 5′-TCC CTG CAT CCA AAG CAC TT; and mitochondrial 39S ribosomal protein L27 (MRPL-27), 5′-CTG GTG GCT GGA ATT GAC CGC TA, and 5′-CAA GGG GAT ATC CAC AGA GTA CCT TG.

### 2.8. Statistical Analysis

Values in bar charts are presented as the mean ± SD of three independent experiments. Statistical comparisons were performed using unpaired Student’s *t*-test if two experimental groups were studied and using one-way analysis of variance (ANOVA) with GraphPad Prism software (version 6) (GraphPad Software, San Diego, CA, USA) if more than two experimental groups were studied. *p* < 0.05 was considered statistically significant.

## 3. Results

### 3.1. Expression of N-WASP Is Reduced in SCC Compared to Matched Perilesional Controls

**Figure 1:**
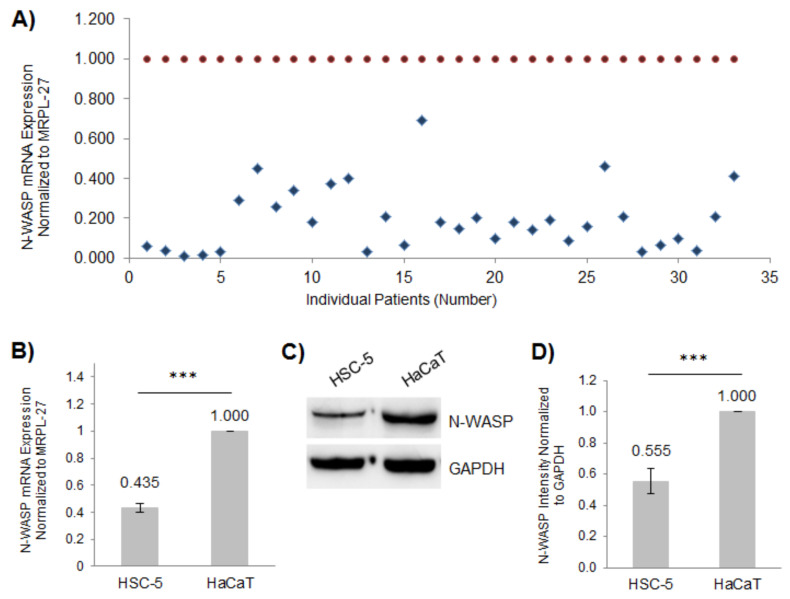
Expression of N-WASP is reduced in SCC patient samples and the SCC cell line HSC-5. (**A**) The total RNA was extracted from the paraffin-embedded SCC and matched perilesional samples and reverse-transcribed to cDNA for quantifying the N-WASP expression relative to MRPL-27 using real-time PCR, normalized to individually matched perilesional samples (SCC values: blue diamonds, matched perilesional values: red circles); *n* = 33. (**B**) The total RNA was extracted from the HSC-5 and HaCaT cells and reverse transcribed to cDNA for quantifying N-WASP expression relative to MRPL-27 using real-time PCR, normalized to HaCaT cells. (**C**) Representative Western blots of N-WASP and GAPDH (loading control) in the HSC-5 and HaCaT cells; *n* = 3. (**D**) Densitometric quantification of the N-WASP/GAPDH ratio in HSC-5 cells normalized to HaCaT cells. All values are the mean ± SD, *n* = 3, *** *p* < 0.001.

To determine the role of N-WASP in skin SCC, we obtained paraffin-embedded SCC samples and matched perilesional samples from 33 skin cancer patients from the National Skin Centre (Singapore). We found that all 33 patient-derived SCC samples had reduced N-WASP expression compared to their matched perilesional controls, as determined by real-time PCR (Figure 1A). The HSC-5 cell line was derived from human skin SCC [38], while the HaCaT cell line is a non-tumorigenic spontaneously immortalized human keratinocyte cell line, often used as a normal skin cell model [39]. We quantified N-WASP mRNA levels in these two cell lines relative to MRPL-27 using real-time PCR. Consistent with the patient SCC, the HSC-5 cells had reduced levels of N-WASP transcript compared to the HaCaT cells (Figure 1B). A Western blot analysis confirmed reduced N-WASP protein levels in the HSC-5 cells compared to the HaCaT cells (Figure 1C,D). These results suggest that the reduced N-WASP plays a role in SCC progression and that the HSC-5 cell line is a suitable model for in vitro studies to characterize the role of N-WASP in skin SCC.

### 3.2. Elevated Expression of N-WASP in HSC-5 Cells Reduced Cell Proliferation and Cell Migration

**Figure 2:**
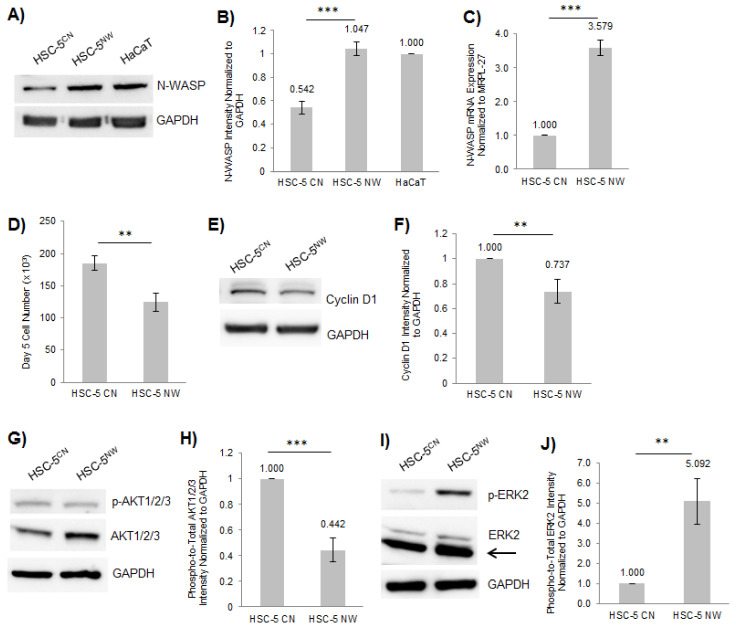
Exogenous expression of N-WASP in HSC-5 cells reduced cell proliferation but increased pERK2 levels. (**A**) Representative Western blots of N-WASP and GAPDH in HSC-5^CN^, HSC-5^NW^ and HaCaT cells; *n* = 3. (**B**) Densitometric quantification of the N-WASP/GAPDH ratio in each cell line normalized to HaCaT cells. (**C**) The total RNA was extracted from HSC-5^CN^ and HSC-5^NW^ cells, reverse-transcribed to cDNA for quantifying N-WASP expression relative to MRPL-27 using real-time PCR and subsequently normalized to HSC-5^CN^ cells. (**D**) The graph shows the total cell number after 5 days of incubation of HSC-5^CN^ and HSC-5^NW^ cells. (**E**,**G**,**I**) Representative Western blots of (**E**) Cyclin D1, (**G**) pan-AKT1/2/3 and pAKT1/2/3, and (**I**) pan-ERK1/2 and pERK1/2, and GAPDH in HSC-5^CN^ and HSC-5^NW^ cells; *n* = 3. (**F**,**H**,**J**) Densitometric quantifications of the ratio of (**F**) cyclin D1/GAPDH, (**H**) pAKT1/2/3/pan-AKT1/2/3, and (**J**) pERK2/pan-ERK2 in HSC-5^CN^ and HSC-5^NW^ cells normalized to HSC-5^CN^ cells. All values are the mean ± SD, *n* = 3, ** *p* < 0.01, *** *p* < 0.001.

SCC samples had a reduced expression of N-WASP (Figure 1A). We previously showed that the conditional ablation of N-WASP expression in mouse keratinocytes increased the proliferation in keratinocytes [15], suggesting that N-WASP may play an inhibitory role in skin carcinogenesis. To this end, we generated two stable HSC-5 cell lines, a control (HSC-5^CN^) and one overexpressing N-WASP (HSC-5^NW^), via infection with lentivirus made using an empty target vector and a vector expressing N-WASP, respectively. Limited dilutions were performed to isolate clonal HSC-5^NW^ cells, and all the resulting clones were analyzed by Western blot to identify a clone that expressed N-WASP at levels similar to that of HaCaT cells (Figure 2A,B). This clone also had higher N-WASP mRNA levels than HSC-5^CN^ cells, as determined by real-time PCR (Figure 2C), implying successful generation of HSC-5^NW^ cells with endogenously elevated N-WASP expression and was used for further studies. However, we observed that N-WASP protein levels in HSC-5^NW^ cells were not proportional to transcript levels (Figure 2B,C), suggesting either posttranscriptional or posttranslational regulation of N-WASP levels in HSC-5 cells.

To characterize the role of N-WASP in cell proliferation, we numerated the total cell number of HSC-5^CN^ and HSC-5^NW^ cells at seeding and after 5 days of incubation. HSC-5^NW^ cells had reduced cell proliferation compared to HSC-5^CN^ cells after 5 days of culture (Figure 2D). Similarly, exogenous expression of N-WASP in A431 and A549 cells also reduced cell proliferation compared to controls (Figure S1), suggesting that N-WASP might suppress SCC tumorigenesis by attenuating cell proliferation. We studied the expression of several proliferative marker proteins in HSC-5^CN^ and HSC-5^NW^ cells via Western blot analysis to confirm the reduced proliferative phenotype. We found that HSC-5^NW^ cells had reduced levels of cyclin D1 (a cell proliferative activity marker [40]) (Figure 2E,F) and reduced levels of active AKT (a protein that stimulates proliferation [41]) (Figure 2G,H) compared to HSC-5^CN^ cells, supporting the hypothesis that N-WASP attenuates cell proliferation. However, we observed higher phospho-ERK2 levels, another cell proliferation indicator [22], in HSC-5^NW^ cells than in HSC-5^CN^ cells (arrow for pan-ERK2) (Figure 2I,J), suggesting that the level of active ERK2 correlates inversely with cell proliferation in HSC-5^NW^ cells.

**Figure 3:**
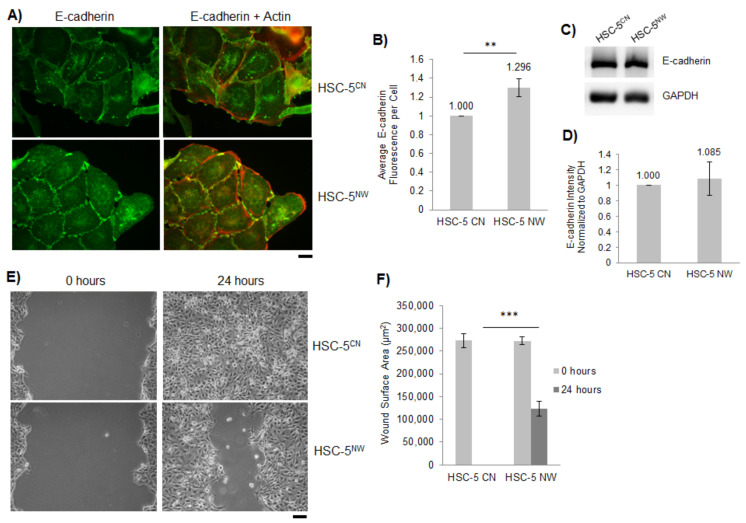
HSC-5^NW^ cells have increased E-cadherin localization and reduced cell migration. (**A**) Representative immunofluorescence images of HSC-5^CN^ and HSC-5^NW^ cells stained for E-cadherin (green). Actin was stained with Alexa Fluor 568 phalloidin (orange–red). Scale bar represents 20 µm, *n* = 3. (**B**) Quantification of E-cadherin fluorescence from 20 randomly chosen cells per experiment based on the number of interacting cell–cell junctions and normalized to HSC-5^CN^ cells. (**C**) Representative Western blots of E-cadherin and GAPDH in HSC-5^CN^ and HSC-5^NW^ cells; *n* = 3. (**D**) Densitometric quantification of the E-cadherin/GAPDH ratio in HSC-5^CN^ and HSC-5^NW^ cells normalized to HSC-5^CN^ cells. Values are the mean ± SD, *n* = 3, *p* > 0.05. (**E**) Representative images of in vitro wounds of HSC-5^CN^ and HSC-5^NW^ cells at 0 and 24 h. Scale bar represents 50 µm, *n* = 3. (**F**) Quantification of wound areas performed in (**E**) at respective time-points using ImageJ. All values are the mean ± SD, *n* = 3, ** *p* <0.01, *** *p* < 0.001.

N-WASP plays a critical role in cell-cell adhesion through a non-canonical post-nucleation pathway [42]. We visualized the localization of E-cadherin, a cell-cell adhesion molecule, via immunofluorescence staining of the HSC-5 sublines. HSC-5^NW^ cells had increased localization of E-cadherin at cell-cell junctions compared to HSC-5^CN^ cells (Figure 3A,B). However, we found that both HSC-5^CN^ and HSC-5^NW^ cells had similar E-cadherin protein expression, as determined by Western blot analysis (Figure 3C,D). This suggests that the increased localization in E-cadherin is not due to increased protein expression; rather, it is due to enhanced E-cadherin localization to the cell-cell junctions as a result of increased N-WASP expression.

We have previously shown that N-WASP inhibits cell migration as N-WASP knockout mouse embryonic fibroblasts (MEFs) migrate faster than wild-type MEFs [43]. Therefore, we performed wound healing assays on HSC-5^CN^ and HSC-5^NW^ cells to investigate differences in cell migration activity in HSC-5 sublines. We found that after 24 h, HSC-5^CN^ cells, not HSC-5^NW^ cells, closed the gap (Figure 3E,F), suggesting that N-WASP attenuates HSC-5 cell migration.

### 3.3. FOXO1 Expression Is Reduced in HSC-5^NW^ Cells Compared to HSC-5^CN^ Cells

**Figure 4:**
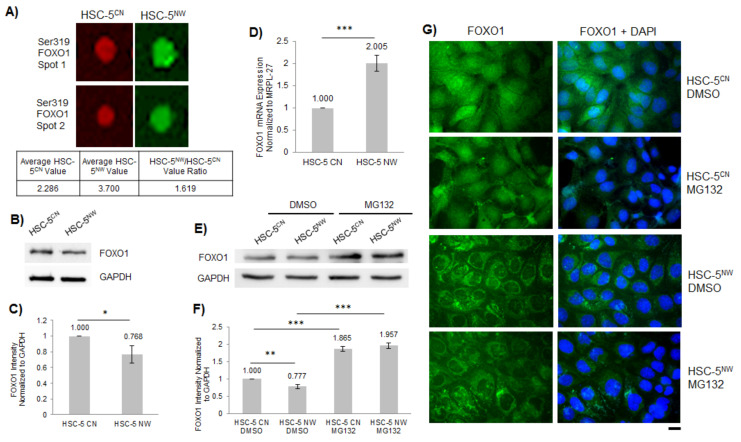
FOXO1 expression is reduced in HSC-5^NW^ cells. (**A**) Individual duplicate arrays of phospho-Ser319 FOXO1 for the HSC-5^CN^ (red) and HSC-5^NW^ (green) cell lysate protein microarrays; the complete Kinex KAM-880 microarray is shown in Figure S2. The average values of HSC-5^CN^ and HSC-5^NW^ cell phospho-Ser319 FOXO1 arrays are shown. (**B**) Representative Western blots of FOXO1 and GAPDH in HSC-5^CN^ and HSC-5^NW^ cells; *n* = 3. (**C**) Densitometric quantification of the FOXO1/GAPDH ratio in HSC-5^CN^ and HSC-5^NW^ cells normalized to HSC-5^CN^ cells. (**D**) Total RNA was extracted from HSC-5^CN^ and HSC-5^NW^ cells and reverse-transcribed to cDNA for quantifying FOXO1 expression relative to MRPL-27 using real-time PCR, normalized to HSC-5^CN^ cells. (**E**) Representative Western blots of FOXO1 and GAPDH in HSC-5^CN^ and HSC-5^NW^ cells treated with 10 µM MG132 or DMSO; *n* = 3. (**F**) Densitometric quantification of the FOXO1/GAPDH ratio in HSC-5^CN^ and HSC-5^NW^ cells treated as in (**E**) normalized to DMSO-treated HSC-5^CN^ cells. (**G**) Representative immunofluorescence images of HSC-5^CN^ and HSC-5^NW^ cells treated as in (**E**) stained for FOXO1 (green). Nuclei were counterstained with DAPI (blue). Scale bar represents 20 µm, *n* = 3. All values are the mean ± SD, *n* = 3, * *p* < 0.05, ** *p* < 0.01, ***p < 0.001.

To identify the signaling pathways altered in HSC-5^NW^ cells, we used the Kinex KAM-880 microarray kit, which detects 518 pan-specific proteins and 359 phospho-specific proteins critical for cell signaling pathways. Cell lysates from HSC-5^CN^ and HSC-5^NW^ cells were labelled with fluorophores and incubated on the Kinex antibody microarray, and the respective microarrays were visualized (Figure S2). The fluorescence intensity measurement for each target protein was based on the average of two duplicate measurements. We considered HSC-5^NW^/HSC-5^CN^ ratio values ≥1.5 to be upregulated in HSC-5^NW^ cells and HSC-5^NW^/HSC-5^CN^ ratio values ≤ 0.5 to be downregulated in HSC-5^NW^ cells. We found that five proteins were downregulated, and thirteen proteins were upregulated in HSC-5^NW^ cells compared to HSC-5^CN^ cells (Table S1). One of the upregulated proteins was phospho-Ser319 FOXO1 (Figure 4A), suggesting that increased phosphorylated FOXO1 is responsible for the reduced proliferation of HSC-5^NW^ cells.

To validate the protein microarray findings, we performed Western blot analysis and real-time PCR on HSC-5^CN^ and HSC-5^NW^ cells. Western blot analysis showed that compared to HSC-5^CN^ cells, HSC-5^NW^ cells had reduced FOXO1 protein levels (Figure 4B,C). However, real-time PCR indicated that HSC-5^NW^ cells had higher FOXO1 mRNA levels than HSC-5^CN^ cells (Figure 4D). The increased mRNA levels in HSC-5^NW^ cells are probably a compensatory mechanism to ensure the minimum FOXO1 protein levels necessary for the proliferation of HSC-5^NW^ cells.

FOXO1 expression in the cell is known to be post-translationally regulated by proteasomal degradation after phosphorylation by several kinases [20,22], which could be responsible for the reduced FOXO1 protein levels in HSC-5^NW^ cells. We performed immunofluorescence staining for FOXO1 in the HSC-5 sublines to determine differences in FOXO1 localizations. Immunofluorescence staining showed that HSC-5^NW^ cells had reduced nuclear staining for FOXO1 compared to HSC-5^CN^ cells (Figure 4G, DMSO), suggesting that N-WASP enhanced the nuclear export of FOXO1. To determine whether the reduced FOXO1 in HSC-5^NW^ cells is due to proteasomal degradation of FOXO1, we treated HSC-5^CN^ and HSC-5^NW^ cells with 10 µM of the proteasome inhibitor benzyl [(2S)-4-methyl-1-{[(2S)-4-methyl-1-{[(2S)-4-methyl-1-oxopentan-2-yl]amino}-1-oxopentan-2-yl]amino}-1-oxopentan-2-yl]carbamate (MG132) or dimethyl sulfoxide (DMSO) (control). Western blot analysis of whole-cell lysates revealed that MG132-treated HSC-5^CN^ and HSC-5^NW^ cells had higher FOXO1 protein levels than DMSO-treated cells (Figure 4E,F). Immunofluorescence staining showed that although MG132 treatment stabilized FOXO1 protein levels, it did not increase nuclear staining in FOXO1 in HSC-5^NW^ cells (Figure 4G), suggesting that MG132 stabilized total FOXO1 levels by inhibiting proteasomal degradation, however, not the nuclear export of FOXO1. These results suggest that increased N-WASP might promote FOXO1 phosphorylation and nuclear export to the cytoplasm for proteasomal degradation, reducing FOXO1 levels in HSC-5^NW^ cells.

### 3.4. Inhibition of ERK2 in HSC-5^NW^ Cells Enhanced Cell Proliferation, Nuclear Localization of FOXO1 and Cell Migration

**Figure 5:**
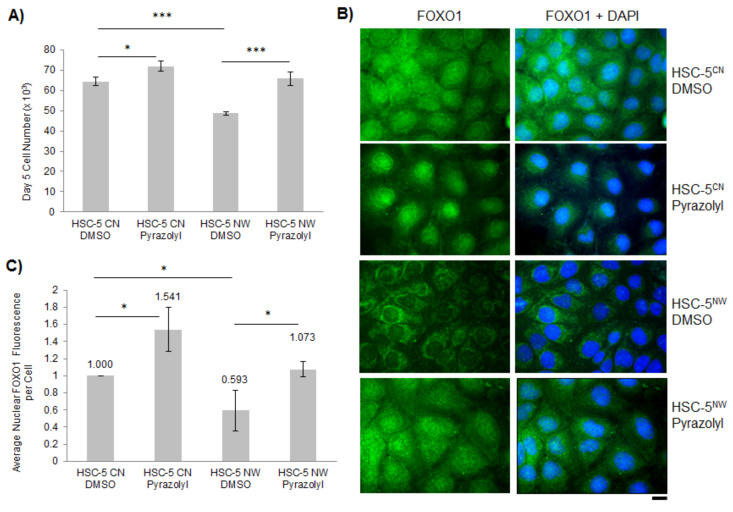
Inhibition of ERK2 in HSC-5^NW^ cells restored cell proliferation and stabilized nuclear FOXO1. (**A**) The graph shows the total cell number after 5 days of incubation of HSC-5^CN^ and HSC-5^NW^ cells treated with 2 nM Pyrazolyl or DMSO. (**B**) Representative immunofluorescence images of HSC-5^CN^ and HSC-5^NW^ cells treated as in (**A**) stained for FOXO1 (green). Nuclei were counterstained with DAPI (blue). Scale bar represents 20 µm, *n* = 3. (**C**) Quantification of nuclear FOXO1 fluorescence from 20 randomly chosen cells per experiment and normalized to DMSO-treated HSC-5^CN^ cells. All values are the mean ± SD, *n* = 3, * *p* < 0.05, *** *p* < 0.001.

ERK2 kinase phosphorylates FOXO1 at multiple residues in NIH3T3 cells [22]. An increase in ERK2 activity is generally correlated with increased cell proliferation [44,45]. However, we found earlier that HSC-5^NW^ cells had increased phospho-ERK2 levels, yet reduced proliferation compared to HSC-5^CN^ cells (Figure 2D,I,J), suggesting that phospho-ERK2 levels correlate inversely with cell proliferation in HSC-5^NW^ cells. To determine the role of ERK2 in HSC-5 cell proliferation, we performed a cell proliferation assay of HSC-5^CN^ and HSC-5^NW^ cells with 2 nM of the ERK2-specific inhibitor Pyrazolylpyrrole ERK inhibitor (Pyrazolyl) or DMSO (control). Pyrazolyl-treated HSC-5^NW^ cells had higher cell proliferation than DMSO-treated HSC-5^NW^ cells and were comparable to DMSO-treated HSC-5^CN^ cells (Figure 5A), suggesting that the inhibition of ERK2 corrected the cell proliferation defect of HSC-5^NW^ cells.

To determine whether the inhibition of ERK2 in HSC-5 cells affects the cellular localization of FOXO1, we performed immunofluorescence staining for FOXO1 in DMSO- or Pyrazolyl-treated HSC-5 sublines. We found that Pyrazolyl-treated HSC-5^NW^ cells had more nuclear FOXO1 staining than DMSO-treated HSC-5^NW^ cells and were similar to DMSO-treated HSC-5^CN^ cells (Figure 5B,C). We also observed that Pyrazolyl-treated HSC-5^CN^ cells had reduced cytoplasmic FOXO1 staining compared to DMSO-treated HSC-5^CN^ cells (Figure 5B). Taken together, these results suggest that ERK2 either phosphorylates FOXO1 or regulates the phosphorylation of FOXO1 in HSC-5^NW^ cells, causing the nuclear export of FOXO1. We believe that ERK2/FOXO1 interaction regulates HSC-5 cell proliferation.

We performed immunofluorescence staining of E-cadherin in DMSO- and Pyrazolyl-treated HSC-5^CN^ and HSC-5^NW^ cells to determine whether ERK2 inhibition affects E-cadherin localization in HSC-5 cells. Pyrazolyl-treated HSC-5^NW^ cells had reduced junctional E-cadherin compared to DMSO-treated HSC-5^NW^ cells, although they were similar to DMSO-treated HSC-5^CN^ cells (Figure S3A,B). Western blot analysis did not show any differences in the protein levels of E-cadherin (Figure S3C) between HSC-5 sublines (*p* > 0.05). These results suggest that ERK2 activity correlates with E-cadherin localization and that the changes in these cellular localizations are not due to differences in protein expression. We performed wound healing assays of these HSC-5 sublines to determine whether ERK2 inhibition affects HSC-5 cell migration with and without 5 µM of the antiproliferative drug cytosine arabinoside (AraC). We found that Pyrazolyl-treated HSC-5^CN^ cells migrated and closed the gap the fastest, followed by DMSO-treated HSC-5^CN^ cells and Pyrazolyl-treated HSC-5^NW^ cells, which migrated at similar rates, while DMSO-treated HSC-5^NW^ cells closed the gap the slowest (Figure S4A,B). We found a similar pattern of migration when the wound healing assay was performed without AraC (Figure S4C,D). These results suggest that ERK2 activity correlates inversely to HSC-5 cell migration and that ERK2-dependent regulation of FOXO1 is responsible for HSC-5 cell proliferation and migration, and that cell migration is independent of cell proliferation, yet both are regulated by ERK2, FOXO1 and N-WASP in HSC-5 cells.

### 3.5. Knockdown of TXNIP in HSC-5^NW^ Cells Restored Cell Proliferation and Migration

**Figure 6:**
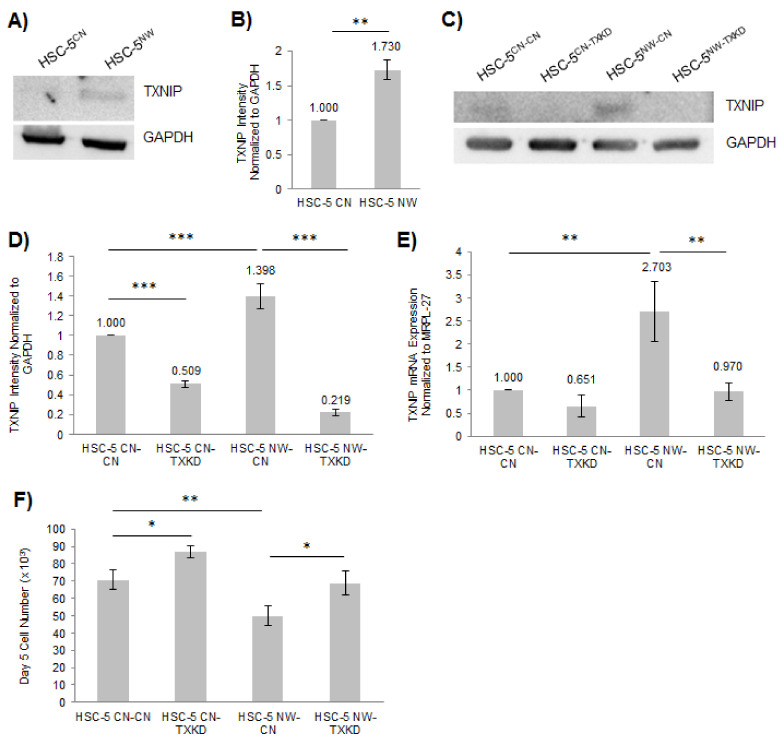
Knockdown of TXNIP expression in HSC-5^NW^ cells enhanced cell proliferation. (**A**) Representative Western blots of TXNIP and GAPDH in HSC-5^CN^ and HSC-5^NW^ cells; *n* = 3. (**B**) Densitometric quantification of the TXNIP/GAPDH ratio in HSC-5^CN^ and HSC-5^NW^ cells normalized to HSC-5^CN^ cells. (**C**) Representative Western blots of TXNIP and GAPDH in HSC-5^CN-CN^, HSC-5^CN-TXKD^, HSC-5^NW-CN^ and HSC-5^NW-TXKD^ cells; *n* = 3. (**D**) Densitometric quantification of the TXNIP/GAPDH ratio in HSC-5^CN-CN^, HSC-5^CN-TXKD^, HSC-5^NW-CN^ and HSC-5^NW-TXKD^ cells normalized to HSC-5^CN-CN^ cells. (**E**) Total RNA was extracted from HSC-5^CN-CN^, HSC-5^CN-TXKD^, HSC-5^NW-CN^ and HSC-5^NW-TXKD^ cells and reverse-transcribed to cDNA for quantifying TXNIP expression relative to MRPL-27 using real-time PCR, normalized to HSC-5^CN-CN^ cells. (**F**) The graph shows the total cell number after 5 days of incubation of HSC-5^CN-CN^, HSC-5^CN-TXKD^, HSC-5^NW-CN^ and HSC-5^NW-TXKD^ cells. All values are the mean ± SD, *n* = 3, * *p* < 0.05, ** *p* < 0.01, *** *p* < 0.001.

FOXO proteins are known to counteract ROS-induced oxidative stress caused by exposure to UV radiation and various environmental factors in skin cells [23]. TXNIP negatively regulates thioredoxin system-mediated ROS-scavenging, and its expression is negatively regulated by FOXO1 [29,31]. The reduced cell proliferation in HSC-5^NW^ cells (Figure 2D) may be due to changes in TXNIP expression and activity. We investigated the role of TXNIP in HSC-5 cell proliferation by performing Western blot analysis of HSC-5^CN^ and HSC-5^NW^ cell lysates. We found that HSC-5^NW^ cells had higher TXNIP expression than HSC-5^CN^ cells (Figure 6A,B). This finding suggests that N-WASP levels correlate with TXNIP levels in HSC-5 cells. To characterize the role of TXNIP in HSC-5^CN^ and HSC-5^NW^ cells, we generated four cell lines by infecting the HSC-5^CN^ and HSC-5^NW^ cell lines with either control lentivirus to generate HSC-5^CN-CN^ and HSC-5^NW-CN^ cells or lentivirus expressing TXNIP-specific shRNA to generate HSC-5^CN-TXKD^ and HSC-5^NW-TXKD^ cells. Western blot analysis of the four sublines revealed a knockdown of TXNIP in HSC-5^CN-TXKD^ and HSC-5^NW-TXKD^ cells compared to HSC-5^CN-CN^ and HSC-5^NW-CN^ cells, respectively (Figure 6C,D). The observed difference in TXNIP protein levels of HSC-5^NW^ and HSC-5^NW-CN^ cells (Figure 6B,D) is probably due to HSC-5^NW-CN^ cells being infected twice with lentiviral infections compared to one lentiviral infection in HSC-5^NW^ cells. However, HSC-5^NW-CN^ cells still had higher TXNIP expression than HSC-5^CN-CN^ cells (Figure 6D). Real-time PCR of the four sublines showed reduced TXNIP mRNA levels in HSC-5^NW-TXKD^ cells compared to HSC-5^NW-CN^ cells (Figure 6E). We observed that TXNIP protein levels in HSC-5^NW^ sublines were not proportional to transcript levels (Figure 6D,E), suggesting either posttranscriptional or posttranslational regulation of TXNIP levels in HSC-5 cells. Cell proliferation assays of the four sublines showed that the cell proliferation of HSC-5^NW-TXKD^ cells was higher than that of HSC-5^NW-CN^ cells and comparable to that of HSC-5^CN-CN^ cells (Figure 6F). This finding suggests that the knockdown of TXNIP corrected the cell proliferation defect of HSC-5^NW^ cells.

We performed immunofluorescence staining and wound healing assays on all four HSC-5 sublines to investigate whether knockdown of TXNIP affects E-cadherin and cell migration in HSC-5 cells. Immunofluorescence staining revealed that HSC-5^NW-TXKD^ cells had reduced junctional E-cadherin compared to that of HSC-5^NW-CN^ cells and comparable to that of HSC-5^CN-CN^ cells (Figure S5A,B). Similarly, Western blot analysis showed no differences in E-cadherin expression in any of the four sublines (Figure S5C) (*p* > 0.05). These results suggest that TXNIP levels correlate with E-cadherin localization and that the changes in cellular localization are not due to differences in protein expression. Wound healing assays performed with 5 µM AraC (Figure S6A,B) and without AraC (Figure S6C,D) found that HSC-5^CN-TXKD^ cells closed the gap the fastest, followed by HSC-5^NW-TXKD^ and HSC-5^CN-CN^ cells, which migrated at similar rates, and HSC-5^NW-CN^ cells, which closed the gap the slowest. The order of cell migration from fastest to slowest was as follows: HSC-5^CN-TXKD^ > HSC-5^NW-TXKD^ = HSC-5^CN-CN^ > HSC-5^NW-CN^. These results suggest that TXNIP activity correlates inversely with HSC-5 cell migration and cell proliferation, and that cell migration is independent of cell proliferation, yet both are regulated by TXNIP and N-WASP in HSC-5 cells.

## 4. Discussion

N-WASP is critical for actin cytoskeleton remodeling, as it activates the Arp2/3 complex and promotes actin polymerization, which is essential for regulating cellular processes such as cell migration and cell proliferation [6,7,46]. Reduced N-WASP levels have been reported to correlate with poor prognosis in breast and colorectal cancer patients [12,13]. N-WASP has also been implicated in lung cancer metastasis [14]. These results suggest that N-WASP may function as a tumor suppressor in breast and colorectal cancers [12,47]. We found that the expression of N-WASP was reduced in SCC patient samples compared to matched perilesional controls (Figure 1A), and the levels of N-WASP protein and transcripts were reduced in the SCC skin cell line HSC-5 compared to non-tumorigenic cell line HaCaT (Figure 1B–D), suggesting that reduced N-WASP levels in skin cells may promote skin carcinogenesis.

N-WASP is critical for cell adhesion and migration in MEF cells [43], and overexpression of N-WASP reduced cell proliferation of HRT18 and MDA-MB-231 cell lines [12,13]. In this study, we also observed reduced cell proliferation in A431 and A549 cells overexpressing N-WASP (Figure S1). However, the molecular mechanism has not been characterized. Vinculin and paxillin regulate cell-ECM adhesion and cell migration [43], while E-cadherin mediates cell-cell adhesion [42]. We hypothesized that N-WASP regulates HSC-5 cell migration via these molecular effectors. HSC-5^NW^ cells had reduced cell proliferation (Figure 2D), which correlated with reduced cyclin D1 expression and reduced active AKT (Figure 2E–H). HSC-5^NW^ cells had increased junctional E-cadherin (Figure 3A,B) and reduced cell migration (Figure 3E,F). Changes in the cellular locations of E-cadherin were not due to changes in protein levels (Figure 3C,D), suggesting that N-WASP regulates the recruitment of E-cadherin, leading to changes in cell-cell adhesion and cell migration.

FOXO1 has been reported to regulate skin epidermal morphogenesis and repair and regulate basal keratinocyte cell proliferation [23,24]. In this study, we performed protein microarray analysis of HSC-5^CN^ and HSC-5^NW^ cells (Figure S2) and found increased phospho-Ser319 FOXO1 in HSC-5^NW^ cells (Table S1, Figure 4A). HSC-5^NW^ cells had reduced total FOXO1 (Figure 4B,C) but increased FOXO1 transcript levels (Figure 4D). HSC-5^NW^ cells had reduced nuclear staining of FOXO1 and increased cytoplasmic staining in FOXO1 compared to HSC-5^CN^ cells (Figure 4G). MG132 treatment of HSC-5 sublines enhanced FOXO1 levels (Figure 4E,F), suggesting that the reduction in FOXO1 in HSC-5^NW^ cells is due to the nuclear export of phosphorylated FOXO1 to the cytoplasm and subsequent proteasomal degradation [21]. Increased FOXO1 transcripts found in HSC-5^NW^ cells are probably a compensatory mechanism to maintain the minimum FOXO1-dependent signaling necessary for cell growth. These results suggest that increased N-WASP levels caused increased FOXO1 phosphorylation, increased nuclear FOXO1 export and increased proteasomal degradation in FOXO1 in HSC-5 cells, which lead to changes in cell proliferation and migration.

ERK2 phosphorylates FOXO1 in NIH3T3 cells [22], and HSC-5^NW^ cells had higher phospho-ERK2 levels than HSC-5^CN^ cells (Figure 2I,J). In this study, we hypothesized that changes in ERK2 activity in HSC-5^NW^ cells led to reduced cell proliferation and migration. Inhibition of ERK2 in HSC-5^NW^ cells using Pyrazolyl restored cell proliferation (Figure 5A) and nuclear staining of FOXO1 (Figure 5B,C) to those of HSC-5^CN^ cells. Pyrazolyl-treated HSC-5^NW^ cells had reduced junctional E-cadherin (Figure S3A,B) and increased cell migration, even in the presence of the antiproliferative drug AraC (Figure S4), similar to those observed in DMSO-treated HSC-5^CN^ cells. It appears that cell proliferation and migration are regulated independently and that both activities are simultaneously regulated by N-WASP through ERK2 in HSC-5 cells. Changes in E-cadherin localizations were not due to changes in expression (Figure S3C). Taken together, these results suggest that increased N-WASP in HSC-5 cells enhanced ERK2-dependent phosphorylation of FOXO1, reduced nuclear FOXO1, and increased cytoplasmic degradation, and reduced total FOXO1, leading to recruitment of E-cadherin and other molecular effectors downstream that cause reduced cell proliferation, adhesion, and migration.

TXNIP negatively regulates thioredoxin system-mediated ROS-scavenging [29], reduces glucose uptake by promoting GLUT1 receptor endocytosis [30,48] and is repressed by FOXO1 [31]. In vitro, TXNIP overexpression led to increased intracellular ROS and can lead to the inhibition of cell proliferation due to apoptosis [34,35]. In this study, we hypothesized that TXNIP regulates HSC-5 cell proliferation and migration. We found that HSC-5^NW^ cells had higher TXNIP expression than HSC-5^CN^ cells (Figure 6A,B), suggesting that TXNIP represses the proliferation of HSC-5^NW^ cells. To study the role of TXNIP in suppressing cell proliferation, we knocked down TXNIP expression in HSC-5^CN^ and HSC-5^NW^ cells (Figure 6C–E). The reduction in HSC-5 cell proliferation caused by N-WASP overexpression (Figure 2D) and its reversal by TXNIP knockdown (Figure 6F) suggest that TXNIP-regulated ROS play a role in regulating HSC-5 cell proliferation in the presence of increased N-WASP. HSC-5^NW-TXKD^ cells also exhibited localizations of junctional E-cadherin (Figure S5A,B) and cell migration, regardless of the presence of AraC (Figure S6), which are similar to those of HSC-5^CN-CN^ cells. Similarly, changes in E-cadherin localizations were not due to changes in E-cadherin expression (Figure S5C), meaning that TXNIP regulates the recruitment of E-cadherin, cell adhesion and migration. It is possible that reduced cell proliferation in HSC-5^NW^ cells is due to reduced glucose uptake following TXNIP-dependent GLUT1 receptor endocytosis [30,48] or increased ROS following TXNIP-dependent inhibition of thioredoxin [29]. It is possible that changes in glucose uptake and ROS levels also play a role in cell adhesion and migration.

**Figure 7:**
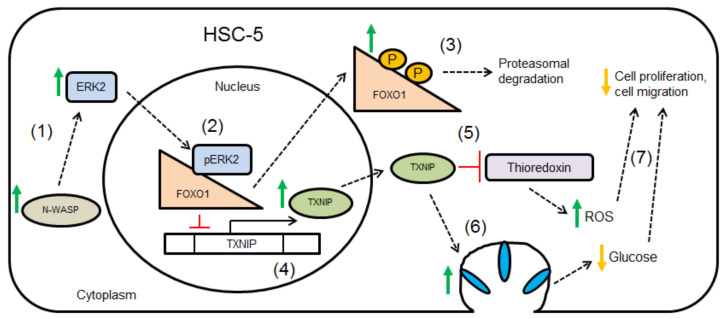
Proposed model for N-WASP-mediated suppression of cell proliferation. (**1**) Increased N-WASP levels enhance ERK2 phosphorylation. (**2**) Active ERK2 translocates from the cytoplasm to the nucleus and causes FOXO1 phosphorylation. (**3**) Phosphorylated FOXO1 translocates from the nucleus to the cytoplasm and is degraded by the proteasome. (**4**) Inhibition of TXNIP expression is relieved. (**5**) TXNIP inhibits the thioredoxin system, causing ROS accumulation. (**6**) TXNIP promotes GLUT1 endocytosis, reducing cellular glucose uptake. (**7**) Changes in ROS levels, glucose levels and activities of molecular effectors lead to decreased cell proliferation and migration.

We [15] and Lyubimova et al. [49] have previously shown that specific knockout of N-WASP results in hyperproliferation of keratinocytes. Reduced expression of N-WASP has been attributed to poor prognosis in breast and colorectal cancer [12,13], probably due to increased cell proliferation and migration. This study suggests that N-WASP regulates ERK2-dependent FOXO1 phosphorylation, TXNIP expression, E-cadherin localization, cell proliferation and migration. Cell proliferation and migration are likely independent of each other, however, they are simultaneously regulated by N-WASP via an ERK2-FOXO1-TXNIP mechanism. The proposed mechanism is visually summarized as shown (Figure 7). Furthermore, increased phospho-ERK2 activity is often correlated with increased proliferation [44,45]; therefore, our results attributing increased phospho-ERK2 with reduced proliferation and the inhibition of ERK2 restoring cell proliferation are novel findings. We propose roles for ERK2, FOXO1 and TXNIP in skin SCC progression as well.

## 5. Conclusions

To summarize, we show that N-WASP negatively regulates cell proliferation and migration through ERK2-dependent FOXO1 phosphorylation, nuclear export, and cytoplasmic proteasomal degradation, leading to TXNIP-mediated changes in these cellular processes. The proposed N-WASP-ERK2-FOXO1-TXNIP pathway may present new avenues for therapeutic intervention in cancer treatment.

## Data Availability

The data presented in this study are available on request from the corresponding author. The data are not publicly available due to restrictions of privacy.

## Supplementary Materials

The following are available online at https://www.mdpi.com/article/10.3390/biology11040582/s1, Figure S1: Exogenous expression of N-WASP in A549 and A431 cells reduced cell proliferation, Figure S2: Protein microarray of HSC-5^CN^ and HSC-5^NW^ cell lysates, Table S1: List of proteins dysregulated in HSC-5^NW^ cells compared to HSC-5^CN^ cells, Figure S3: Inhibition of ERK2 in HSC-5^NW^ cells reduced junctional E-cadherin, Figure S4: Inhibition of ERK2 in HSC-5^NW^ cells enhanced cell migration, Figure S5: Knockdown of TXNIP in HSC-5^NW^ cells reduced junctional E-cadherin, Figure S6: Knockdown of TXNIP in HSC-5^NW^ cells enhanced cell migration.
