# Supplementary material for: N-WASP Attenuates Cell Proliferation and Migration through ERK2-Dependent Enhanced Expression of TXNIP
Source: Biology (Basel). 2022 Apr 11;11(4):582. doi: 10.3390/biology11040582 (PMC9029996; doi:10.3390/biology11040582)
Supplement: Supplementary file 1 [file biology-11-00582-s001.zip › biology-1592350-supplementary.pdf]

biology

MDPI

Supplementary Materials

N-WASP Attenuates Cell Proliferation and Migration through ERK2-Dependent Enhanced Expression of TXNIP

Yat Joong Chung [1], Amrita Salvi [1,†], Pazhanichamy Kalailingam [1], Myra Alnawaz [1], Suat Hoon Tan [2], Jiun Yit Pan [2], Nguan Soon Tan [1,3] and Thirumaran Thanabalu [1,*]

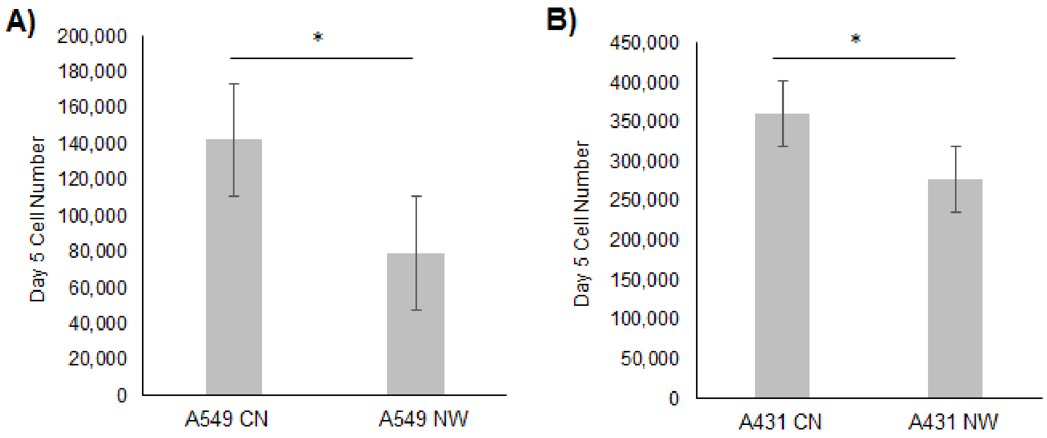

Figure S1. Exogenous expression of N-WASP in A549 and A431 cells reduced cell proliferation. The graphs show the total cell numbers after 5 days of incubation of (**A**) A549^{CN} and A549^{NW} cells, and (**B**) A431^{CN} and A431^{NW} cells. All values are the mean ± SD, $n = 3$, * $p < 0.05$.

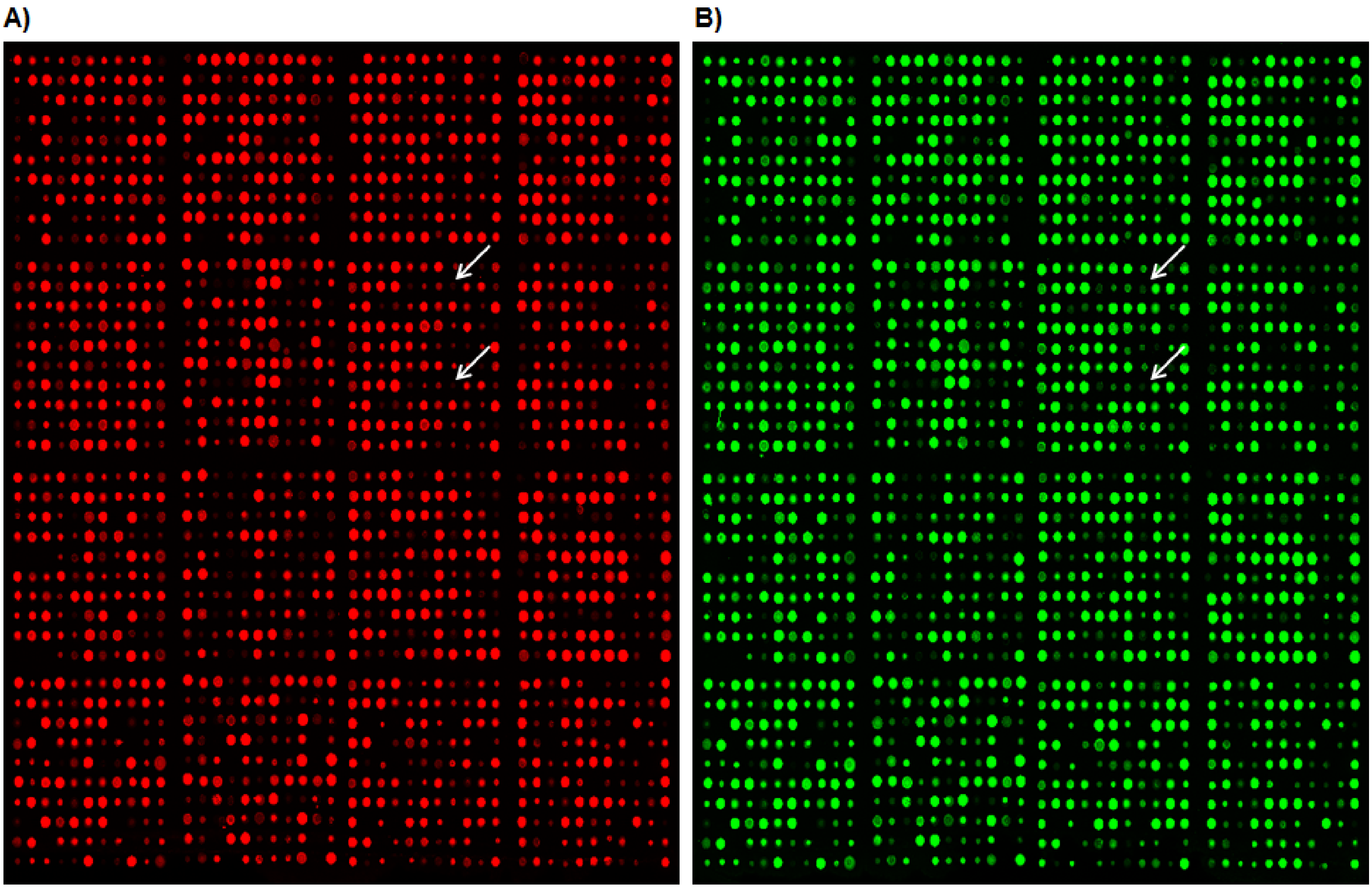

Figure S2. Protein microarray of HSC-5^{CN} and HSC-5^{NW} cell lysates. Labeled protein lysates from (**A**) HSC-5^{CN} (red) and (**B**) HSC-5^{NW} (green) cells were incubated on the Kinex KAM-880 protein microarray, and the microarray was imaged with the Gene Pix Pro 6.0 program. Arrows highlight the duplicate antibody locations for detection and measurement of phospho-Ser319 FOXO1; the magnified images of which are shown in Figure 4A.

Table S1. List of proteins dysregulated in HSC-5^{NW} cells compared to HSC-5^{CN} cells. HSC-5^{NW}/HSC-5^{CN} signal ratio values ≥ 1.5 (up-regulated) and HSC-5^{NW}/HSC-5^{CN} signal ratio values ≤ 0.5 (down-regulated) were considered significant. The target protein names, their respective phosphorylated site, average detection values of HSC-5^{CN} and HSC-5^{NW} cells and the HSC-5^{NW}/HSC-5^{CN} ratio values following the performance of Kinex KAM-880 protein microarray are shown. Phospho-Ser319 FOXO1 is highlighted in blue.

Target Protein Name	Phospho Site (Human)	Full Target Protein Name	HSC-5^CN^ Detection Value	HSC-5^NW^ Detection Value	HSC-5^NW^/HSC-5^CN^ Ratio
p70 S6K	T421/S424	Ribosomal protein S6 kinase beta-1	0.181	0.356	1.970
STAT1	S727	Signal transducer and activator of transcription 1 alpha	2.099	4.081	1.944
BMX (Etk)	Y40	Bone marrow X protein-tyrosine kinase	1.175	2.210	1.881
Met	Y1230/Y1234/ Y1235	Hepatocyte growth factor (HGF) receptor-tyrosine kinase	2.307	4.175	1.809
JNK 1/2/3	T183/Y185	Jun N-terminus protein-serine kinase (stress-activated protein kinase (SAPK)) 1/2/3	2.594	4.613	1.778
4E-BP1	S65	Eukaryotic translation initiation factor 4E binding protein 1 (PHAS1)	1.527	2.570	1.683
RSK1/2/3	T573	Ribosomal S6 protein-serine kinase 1/2/3	1.744	2.905	1.666
Ezrin	T567	Cytovillin 2	1.529	2.535	1.658
FKHR	S319	Forkhead box protein O1	2.286	3.700	1.619
Caveolin 1	Pan-specific	Caveolin 1	1.911	3.042	1.592
Tyrosine Hydroxylase	S40	Tyrosine hydroxylase isoform a	0.429	0.671	1.565
IRS1	S312	Insulin receptor substrate 1	2.532	3.898	1.539
CDK1/2	Y15	Cyclin-dependent protein-serine kinase 1/2	2.210	3.357	1.519
S6K	T412	Ribosomal protein S6 kinase beta-1	0.147	0.004	0.029
PKCl	Pan-specific	Protein-serine kinase C lambda/iota	0.187	0	0
PTPD1	Pan-specific	Protein-tyrosine phosphatase non-receptor type 21	0.222	0	0
RSK1	Pan-specific	Ribosomal S6 protein-serine kinase 1	0.009	0	0
Yes	Pan-specific	Yamaguchi sarcoma proto-oncogene-encoded tyrosine kinase	0.102	0	0

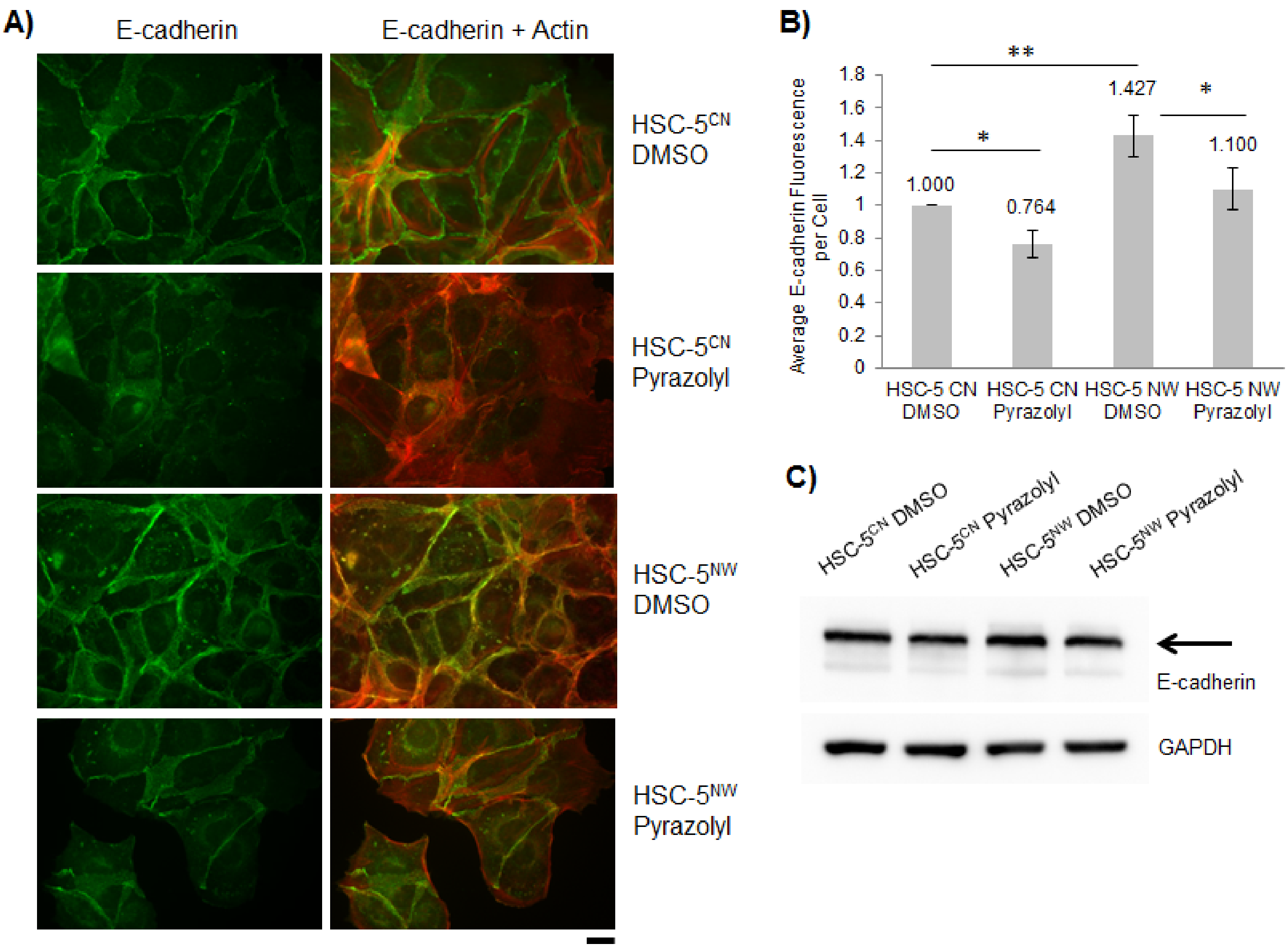

Figure S3. Inhibition of ERK2 in HSC-5^{NW} cells reduced junctional E-cadherin. (**A**) Representative immunofluorescence images of HSC-5^{CN} and HSC-5^{NW} cells treated with 2 nM Pyrazolyl or DMSO stained for E-cadherin (green). Actin was stained with Alexa Fluor 568 phalloidin (orange-red). Scale bar represents 20 µm, n = 3. (**B**) Quantification of E-cadherin fluorescence from 20 randomly chosen cells treated as in (**A**) based on the number of interacting cell-cell junctions and normalized to DMSO-treated HSC-5^{CN} cells. (**C**) Representative Western blots of E-cadherin and GAPDH (loading control) in HSC-5^{CN} and HSC-5^{NW} cells treated as in (**A**); n = 3. All values are the mean ± SD, n = 3, * $p < 0.05$, ** $p < 0.01$.

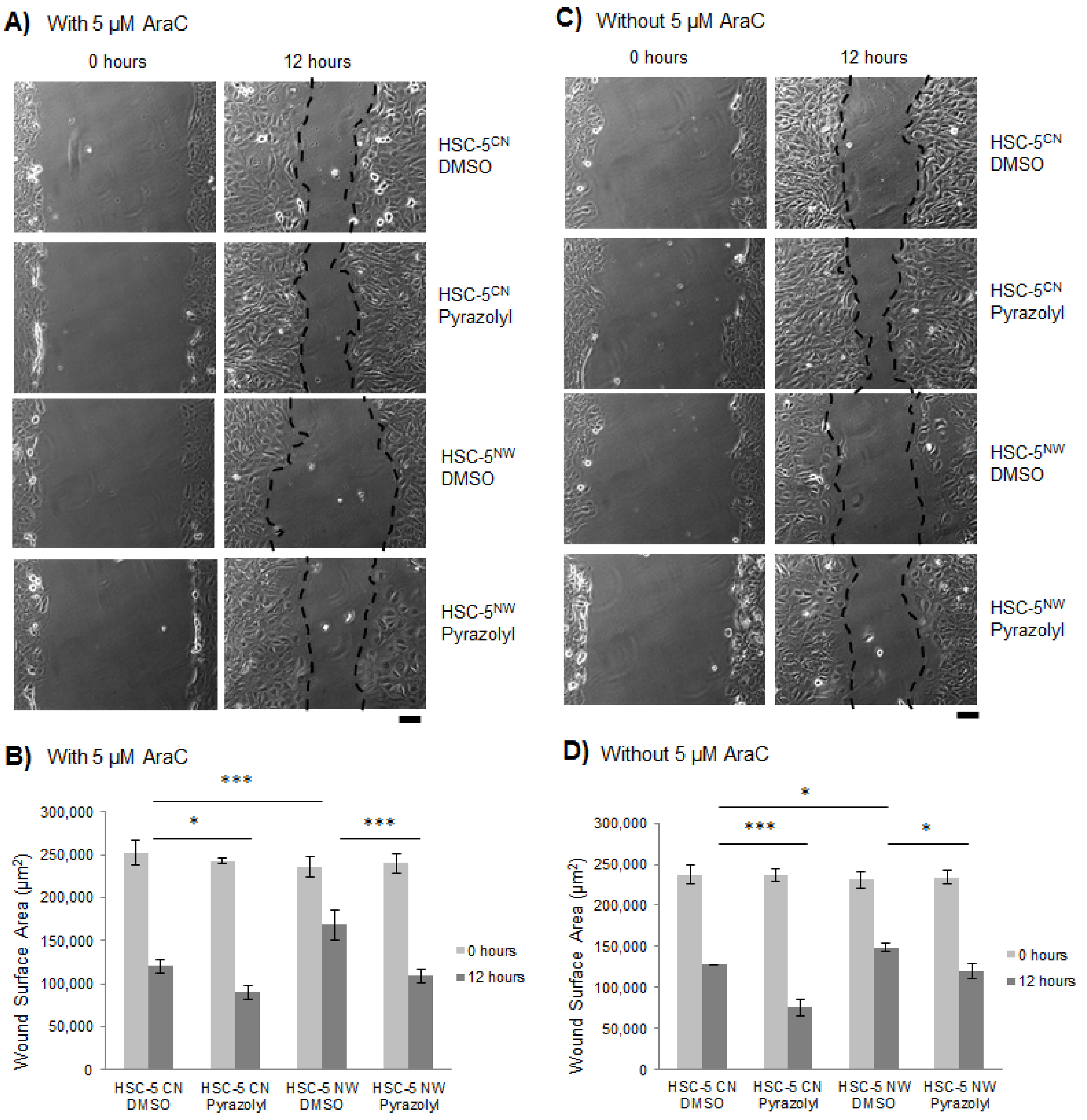

Figure S4. Inhibition of ERK2 in HSC-5^{NW} cells enhanced cell migration. (**A**,**C**) Representative images of in vitro wounds of HSC-5^{CN} and HSC-5^{NW} cells treated with 2 nM Pyrazolyl or DMSO at 0 and 12 h (**A**) with and (**C**) without 5 μM of the antiproliferative drug cytosine arabinoside (AraC). Scale bar represents 50 μm, $n = 3$. (**B**,**D**) Quantification of wound areas in (**A**) and (**C**) respectively using ImageJ. All values are the mean ± SD, $n = 3$, * $p < 0.05$, *** $p < 0.001$.

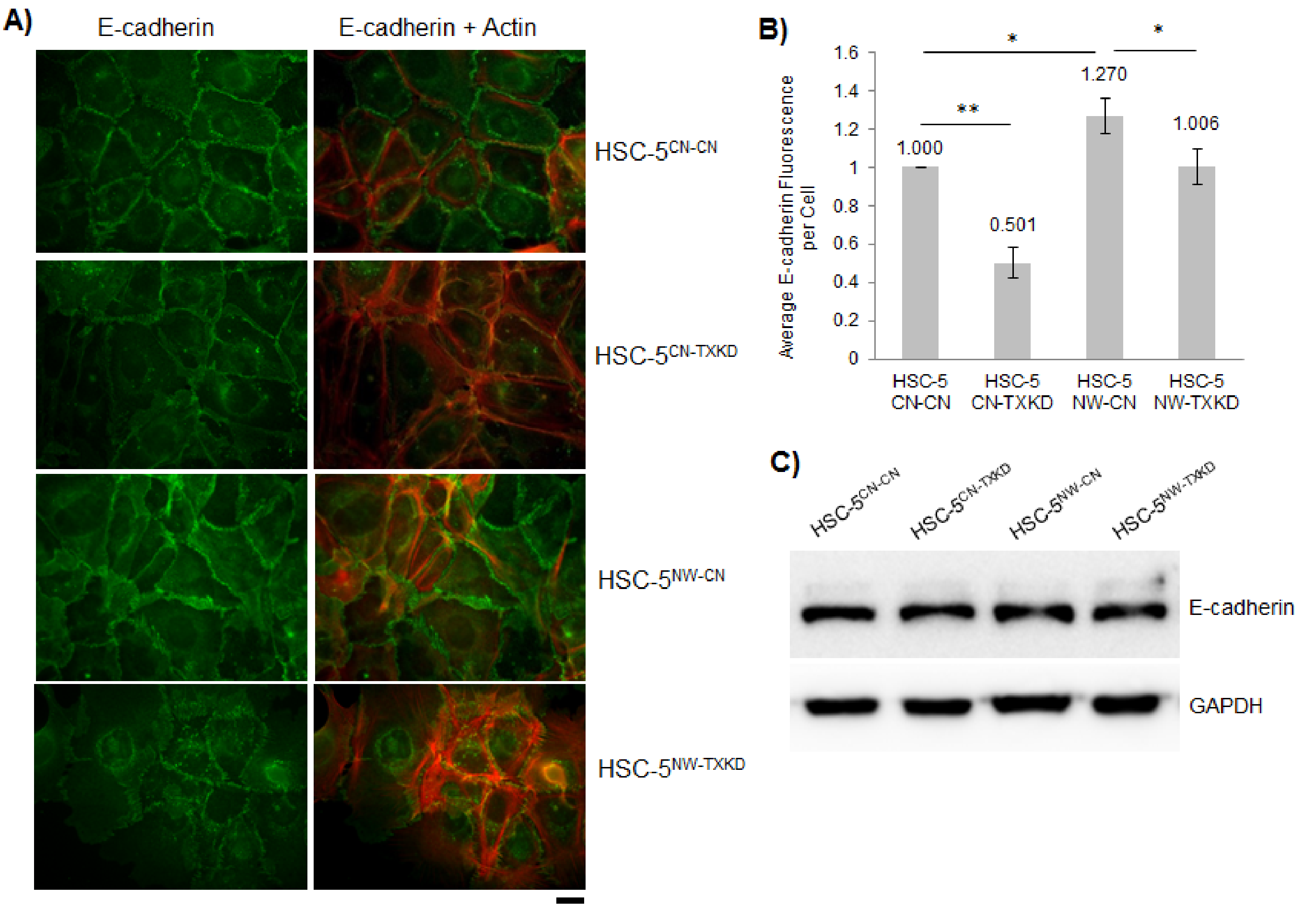

Figure S5. Knockdown of TXNIP in HSC-5^{NW} cells reduced junctional E-cadherin. (**A**) Representative immunofluorescence images of HSC-5^{CN-CN}, HSC-5$^{CN-TXKD}$, HSC-5^{NW-CN} and HSC-5$^{NW-TXKD}$ cells stained for E-cadherin (green). Actin was stained with Alexa Fluor 568 phalloidin (orange-red). Scale bar represents 20 μm, $n = 3$. (**B**) Quantification of E-cadherin fluorescence from 20 randomly chosen cells based on the number of interacting cell-cell junctions and normalized to HSC-5^{CN-CN} cells. (**C**) Representative Western blots of E-cadherin and GAPDH in HSC-5^{CN-CN}, HSC-5$^{CN-TXKD}$, HSC-5^{NW-CN} and HSC-5$^{NW-TXKD}$ cells; $n = 3$. All values are the mean ± SD, $n = 3$, * $p < 0.05$, ** $p < 0.01$.

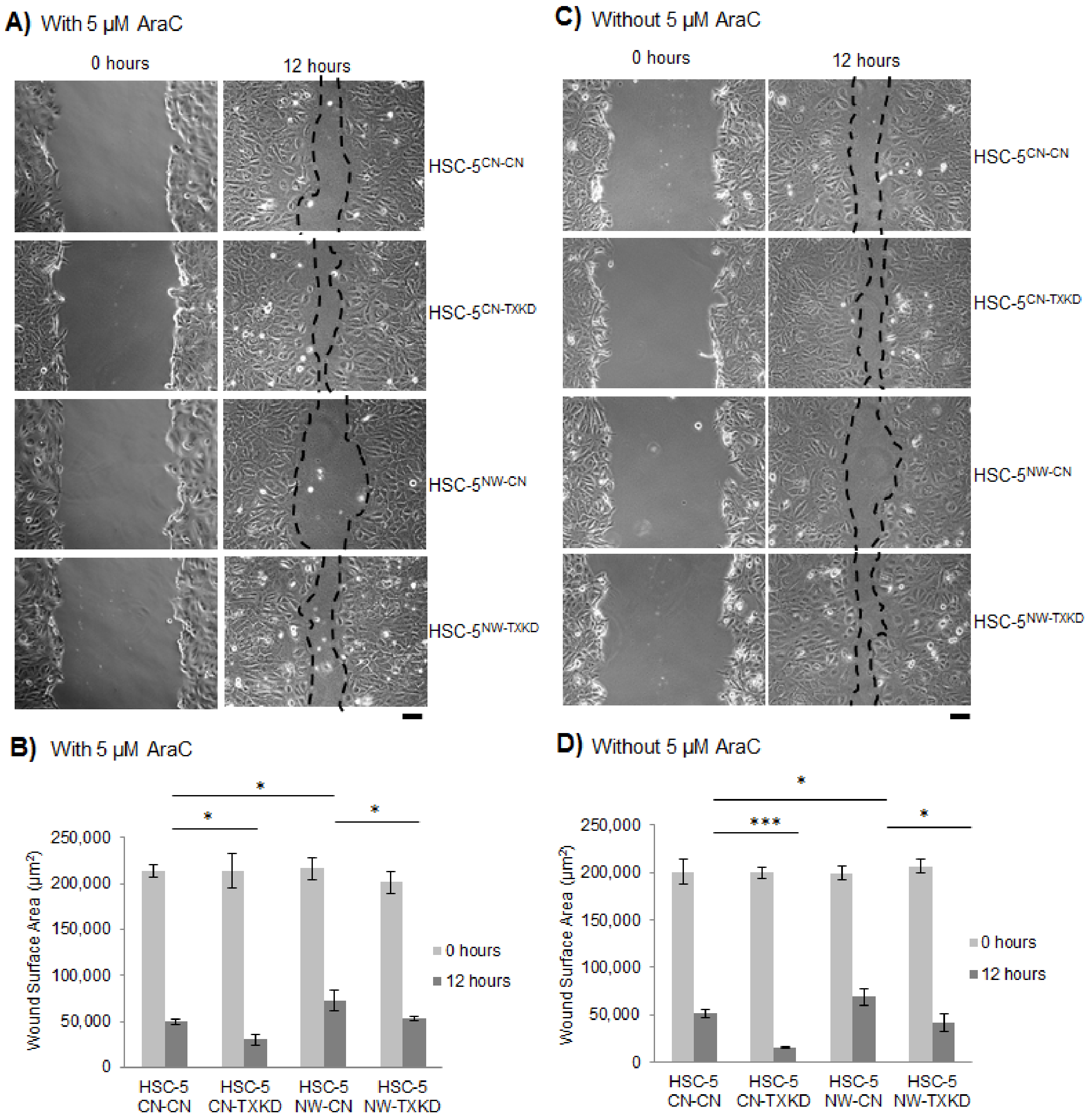

Figure S6. Knockdown of TXNIP in HSC-5^{NW} cells enhanced cell migration. (**A**,**C**) Representative images of in vitro wounds of HSC-5^{CN-CN}, HSC-$5^{CN-TXKD}$, HSC-5^{NW-CN} and HSC-$5^{NW-TXKD}$ cells at 0 and 12 h (**A**) with and (**C**) without 5 μM of AraC. Scale bar represents 50 μm, $n = 3$. (**B**,**D**) Quantification of wound areas in (**A**) and (**C**) respectively using ImageJ. All values are the mean ± SD, $n = 3$, * $p < 0.05$, *** $p < 0.001$
